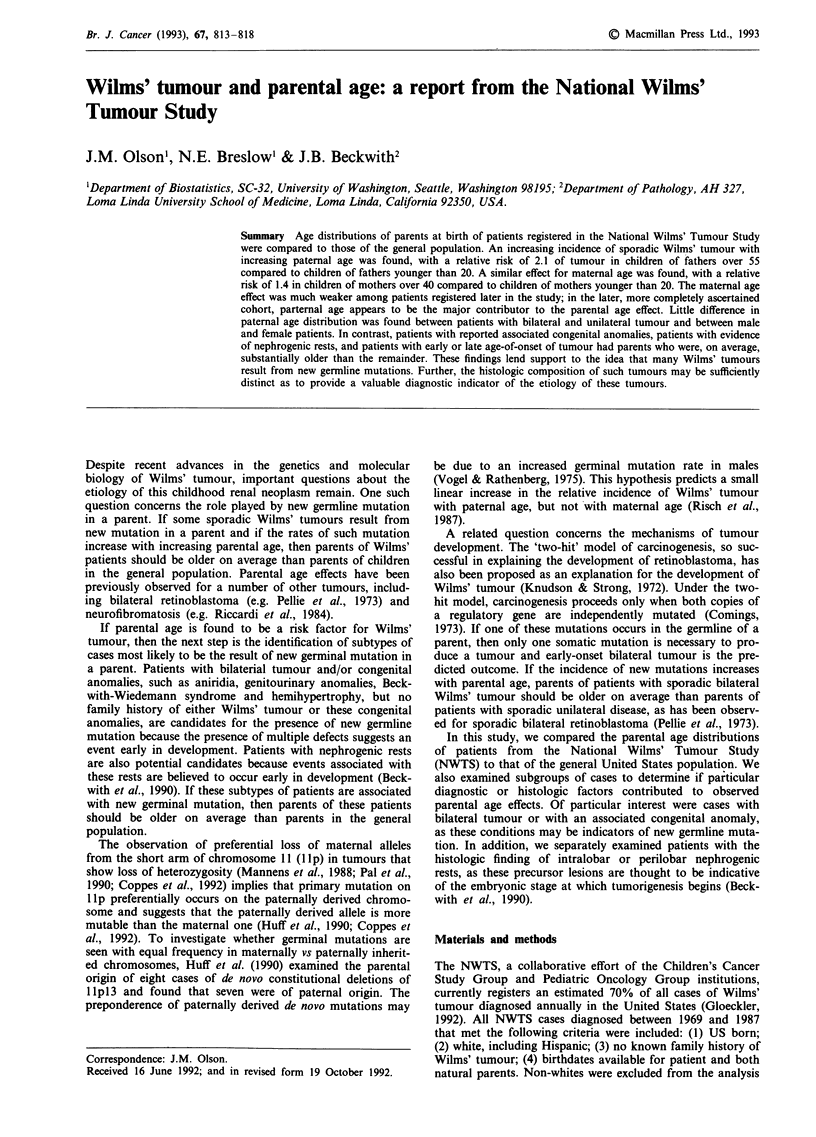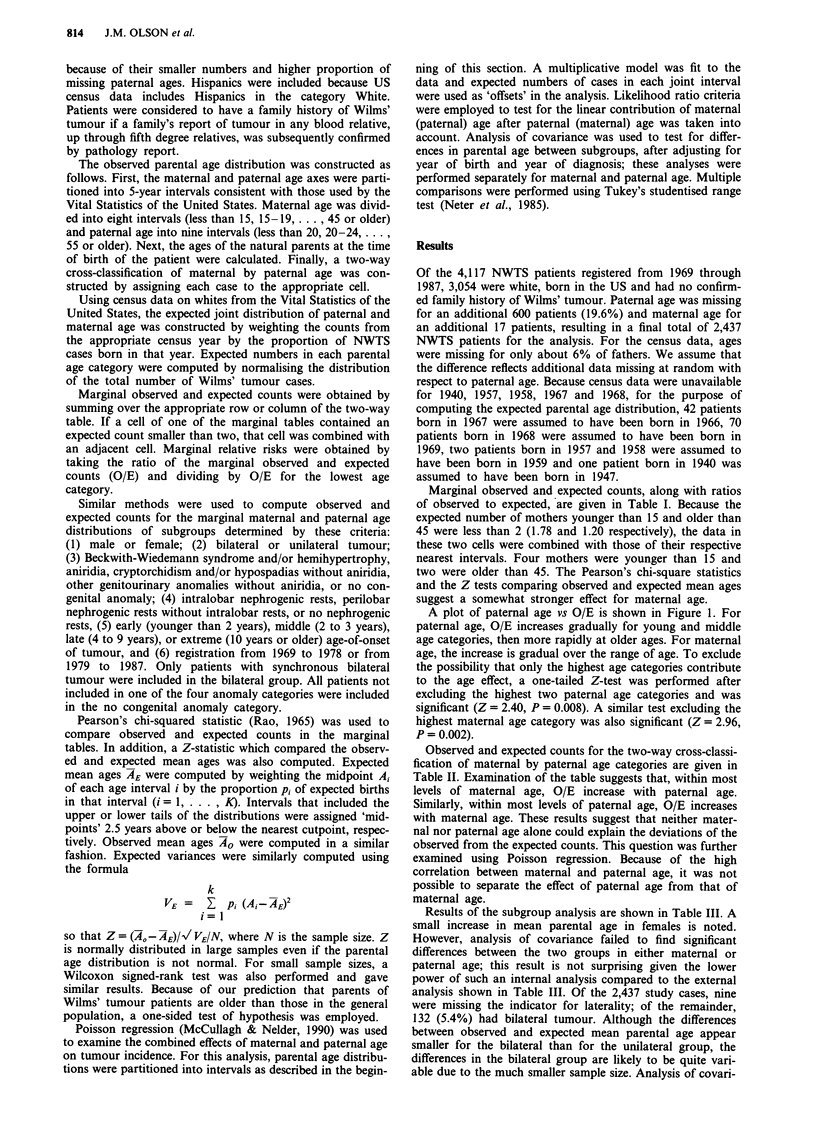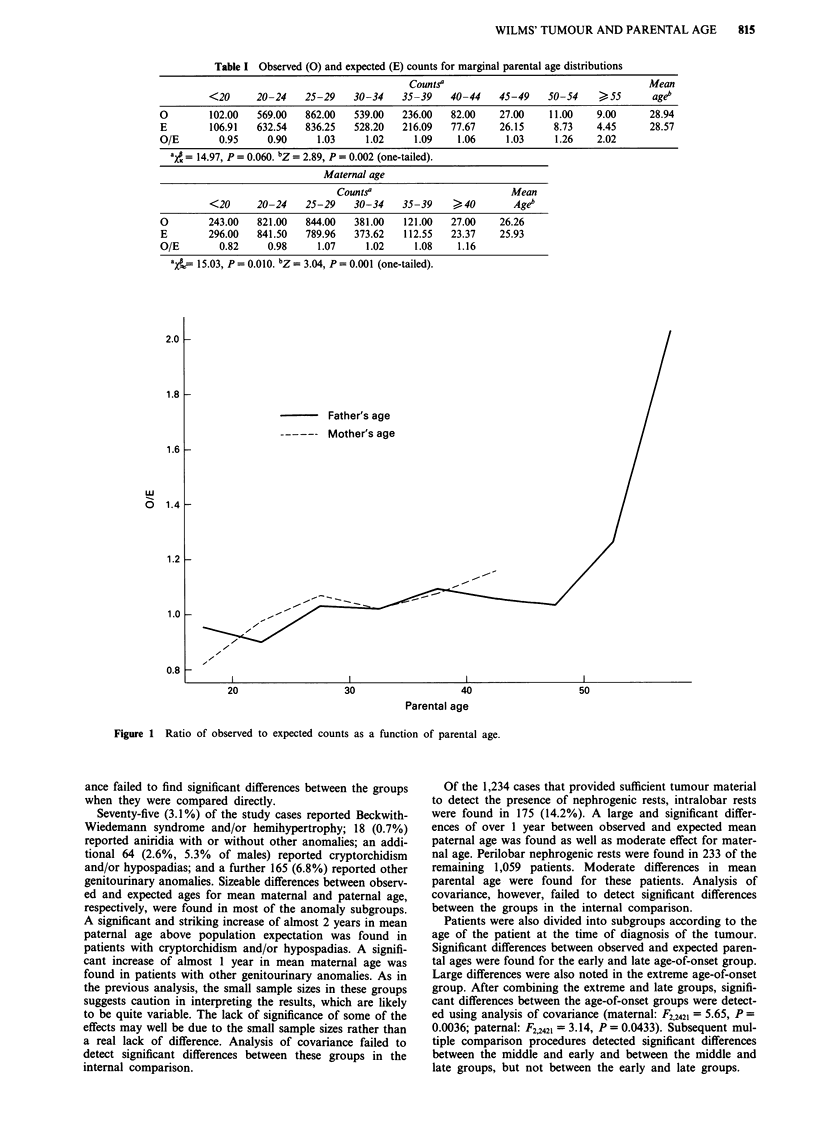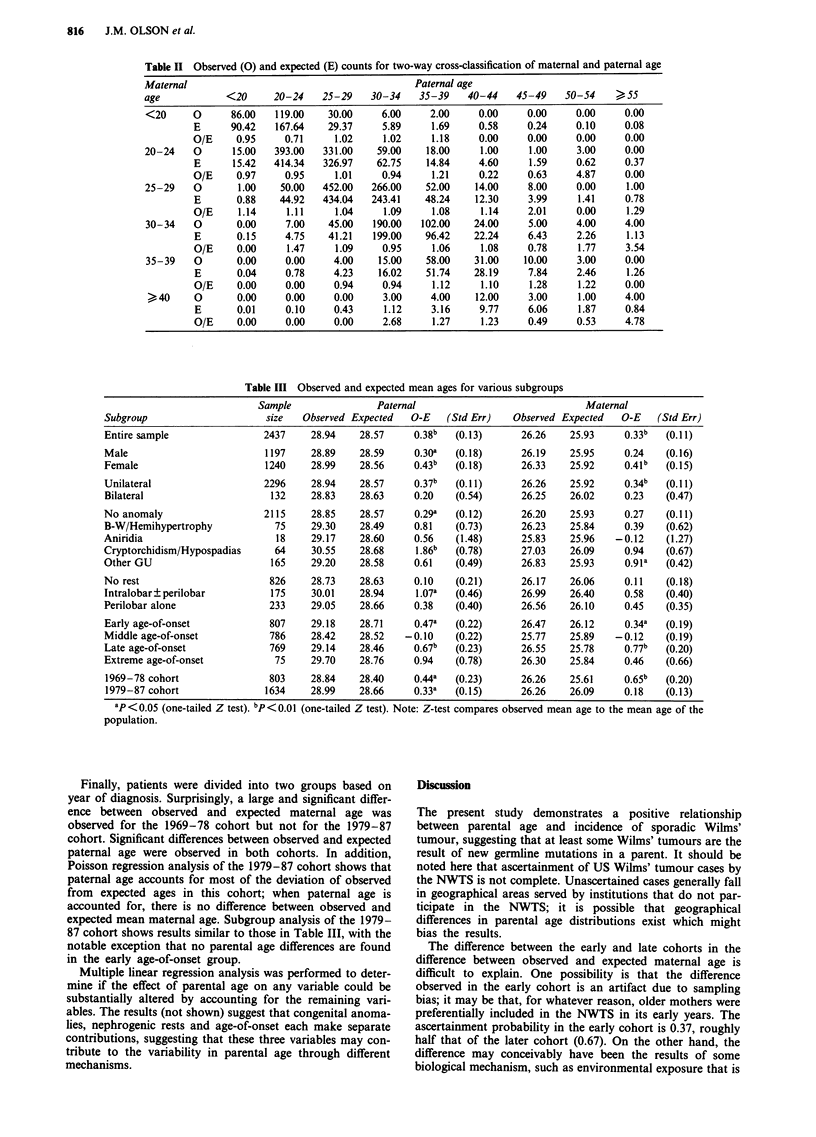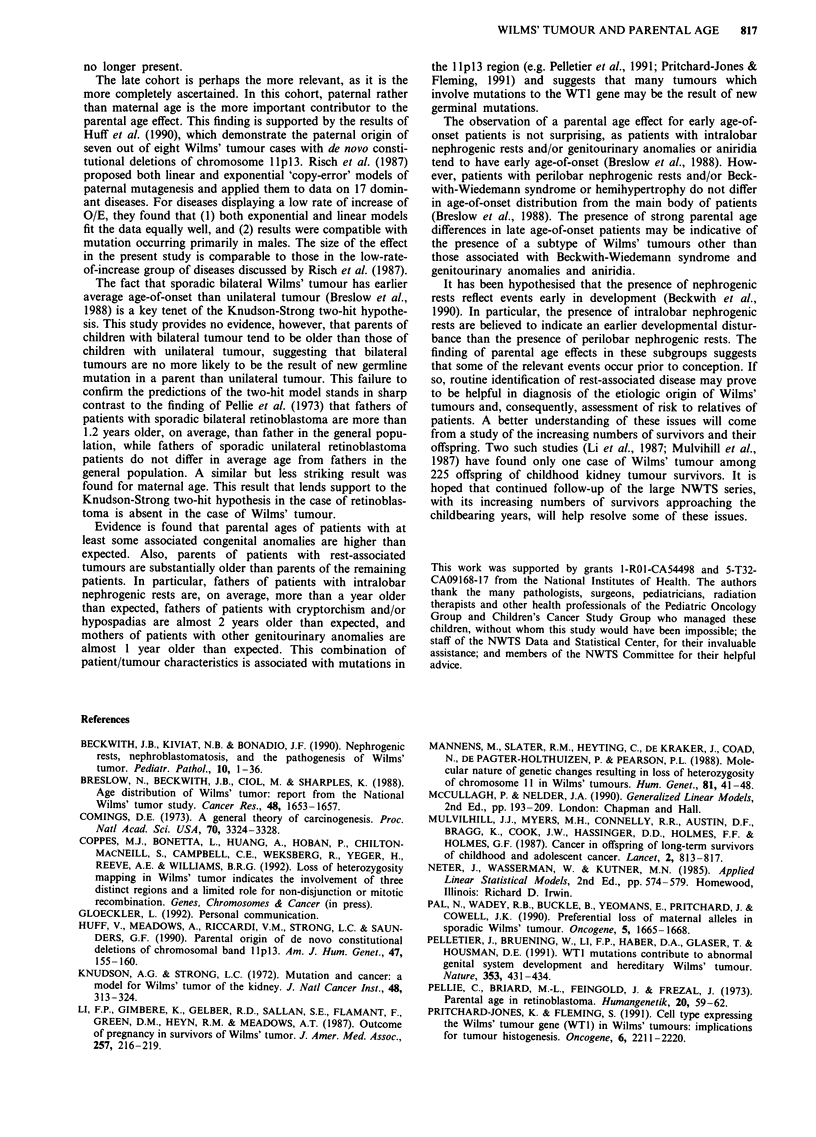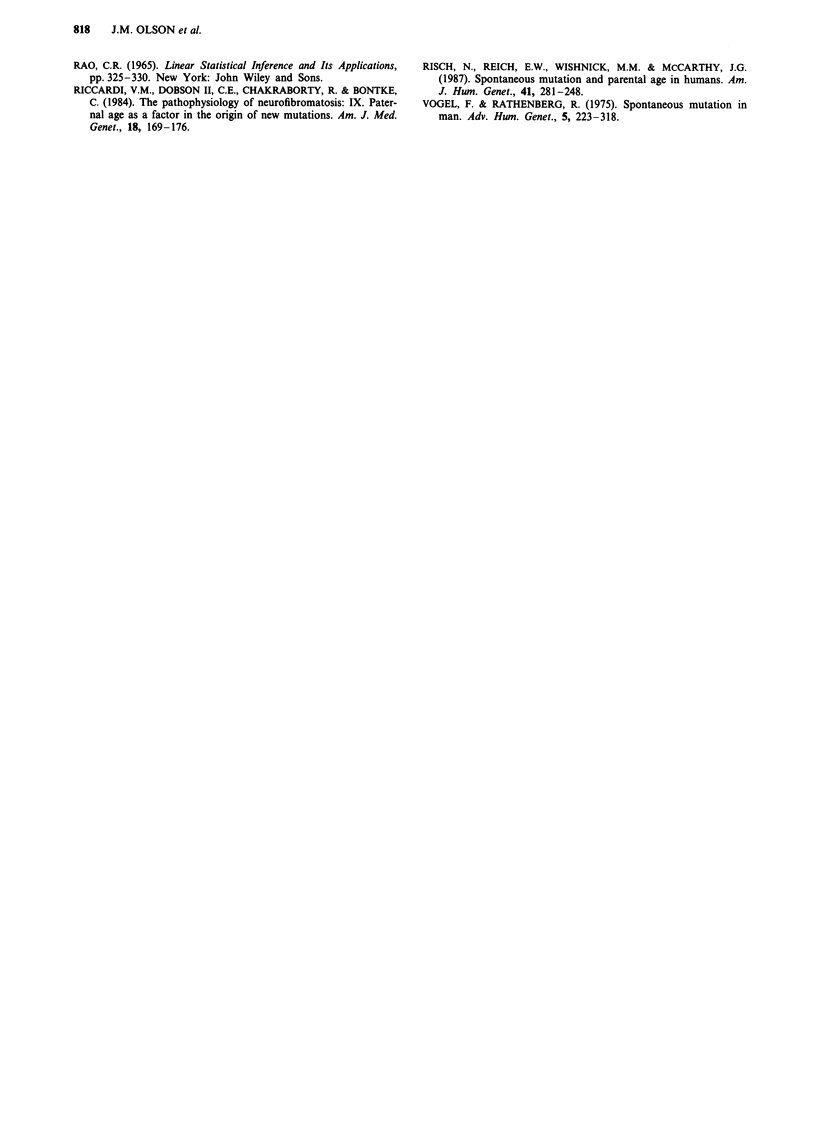# Wilms' tumour and parental age: a report from the National Wilms' Tumour Study.

**DOI:** 10.1038/bjc.1993.148

**Published:** 1993-04

**Authors:** J. M. Olson, N. E. Breslow, J. B. Beckwith

**Affiliations:** Department of Biostatistics, University of Washington, Seattle 98195.

## Abstract

Age distributions of parents at birth of patients registered in the National Wilms' Tumour Study were compared to those of the general population. An increasing incidence of sporadic Wilms' tumour with increasing paternal age was found, with a relative risk of 2.1 of tumour in children of fathers over 55 compared to children of fathers younger than 20. A similar effect for maternal age was found, with a relative risk of 1.4 in children of mothers over 40 compared to children of mothers younger than 20. The maternal age effect was much weaker among patients registered later in the study; in the later, more completely ascertained cohort, paternal age appears to be the major contributor to the parental age effect. Little difference in paternal age distribution was found between patients with bilateral and unilateral tumour and between male and female patients. In contrast, patients with reported associated congenital anomalies, patients with evidence of nephrogenic rests, and patients with early or late age-of-onset of tumour had parents who were, on average, substantially older than the remainder. These findings lend support to the idea that many Wilms' tumours result from new germline mutations. Further, the histologic composition of such tumours may be sufficiently distinct as to provide a valuable diagnostic indicator of the etiology of these tumours.


Br. J. Cancer (1993), 67, 813-818 © Macmillan Press Ltd., 1993

### Wilms' tumour and parental age: a report from the National Wilms' Tumour Study

J.M. Olson[1], N.E. Breslow[1] & J.B. Beckwith[2]

[1]*Department of Biostatistics, SC-32, University of Washington, Seattle, Washington 98195;* [2]*Department of Pathology, AH 327, Loma Linda University School of Medicine, Loma Linda, California 92350, USA.*

**Summary** Age distributions of parents at birth of patients registered in the National Wilms' Tumour Study were compared to those of the general population. An increasing incidence of sporadic Wilms' tumour with increasing paternal age was found, with a relative risk of 2.1 of tumour in children of fathers over 55 compared to children of fathers younger than 20. A similar effect for maternal age was found, with a relative risk of 1.4 in children of mothers over 40 compared to children of mothers younger than 20. The maternal age effect was much weaker among patients registered later in the study; in the later, more completely ascertained cohort, parternal age appears to be the major contributor to the parental age effect. Little difference in paternal age distribution was found between patients with bilateral and unilateral tumour and between male and female patients. In contrast, patients with reported associated congenital anomalies, patients with evidence of nephrogenic rests, and patients with early or late age-of-onset of tumour had parents who were, on average, substantially older than the remainder. These findings lend support to the idea that many Wilms' tumours result from new germline mutations. Further, the histologic composition of such tumours may be sufficiently distinct as to provide a valuable diagnostic indicator of the etiology of these tumours.

Despite recent advances in the genetics and molecular biology of Wilms' tumour, important questions about the etiology of this childhood renal neoplasm remain. One such question concerns the role played by new germline mutation in a parent. If some sporadic Wilms' tumours result from new mutation in a parent and if the rates of such mutation increase with increasing parental age, then parents of Wilms' patients should be older on average than parents of children in the general population. Parental age effects have been previously observed for a number of other tumours, including bilateral retinoblastoma (e.g. Pellie *et al.*, 1973) and neurofibromatosis (e.g. Riccardi *et al.*, 1984).

If parental age is found to be a risk factor for Wilms' tumour, then the next step is the identification of subtypes of cases most likely to be the result of new germinal mutation in a parent. Patients with bilaterial tumour and/or congenital anomalies, such as aniridia, genitourinary anomalies, Beckwith-Wiedemann syndrome and hemihypertrophy, but no family history of either Wilms' tumour or these congenital anomalies, are candidates for the presence of new germline mutation because the presence of multiple defects suggests an event early in development. Patients with nephrogenic rests are also potential candidates because events associated with these rests are believed to occur early in development (Beckwith *et al.*, 1990). If these subtypes of patients are associated with new germinal mutation, then parents of these patients should be older on average than parents in the general population.

The observation of preferential loss of maternal alleles from the short arm of chromosome 11 (11p) in tumours that show loss of heterozygosity (Mannens *et al.*, 1988; Pal *et al.*, 1990; Coppes *et al.*, 1992) implies that primary mutation on 11p preferentially occurs on the paternally derived chromosome and suggests that the paternally derived allele is more mutable than the maternal one (Huff *et al.*, 1990; Coppes *et al.*, 1992). To investigate whether germinal mutations are seen with equal frequency in maternally *vs* paternally inherited chromosomes, Huff *et al.* (1990) examined the parental origin of eight cases of *de novo* constitutional deletions of 11p13 and found that seven were of paternal origin. The preponderence of paternally derived *de novo* mutations may be due to an increased germinal mutation rate in males (Vogel & Rathenberg, 1975). This hypothesis predicts a small linear increase in the relative incidence of Wilms' tumour with paternal age, but not with maternal age (Risch *et al.*, 1987).

A related question concerns the mechanisms of tumour development. The 'two-hit' model of carcinogenesis, so successful in explaining the development of retinoblastoma, has also been proposed as an explanation for the development of Wilms' tumour (Knudson & Strong, 1972). Under the two-hit model, carcinogenesis proceeds only when both copies of a regulatory gene are independently mutated (Comings, 1973). If one of these mutations occurs in the germline of a parent, then only one somatic mutation is necessary to produce a tumour and early-onset bilateral tumour is the predicted outcome. If the incidence of new mutations increases with parental age, parents of patients with sporadic bilateral Wilms' tumour should be older on average than parents of patients with sporadic unilateral disease, as has been observed for sporadic bilateral retinoblastoma (Pellie *et al.*, 1973).

In this study, we compared the parental age distributions of patients from the National Wilms' Tumour Study (NWTS) to that of the general United States population. We also examined subgroups of cases to determine if particular diagnostic or histologic factors contributed to observed parental age effects. Of particular interest were cases with bilateral tumour or with an associated congenital anomaly, as these conditions may be indicators of new germline mutation. In addition, we separately examined patients with the histologic finding of intralobar or perilobar nephrogenic rests, as these precursor lesions are thought to be indicative of the embryonic stage at which tumorigenesis begins (Beckwith *et al.*, 1990).

### Materials and methods

The NWTS, a collaborative effort of the Children's Cancer Study Group and Pediatric Oncology Group institutions, currently registers an estimated 70% of all cases of Wilms' tumour diagnosed annually in the United States (Gloeckler, 1992). All NWTS cases diagnosed between 1969 and 1987 that met the following criteria were included: (1) US born; (2) white, including Hispanic; (3) no known family history of Wilms' tumour; (4) birthdates available for patient and both natural parents. Non-whites were excluded from the analysis

Correspondence: J.M. Olson.
Received 16 June 1992; and in revised form 19 October 1992.

because of their smaller numbers and higher proportion of missing paternal ages. Hispanics were included because US census data includes Hispanics in the category White. Patients were considered to have a family history of Wilms' tumour if a family's report of tumour in any blood relative, up through fifth degree relatives, was subsequently confirmed by pathology report.

The observed parental age distribution was constructed as follows. First, the maternal and paternal age axes were partitioned into 5-year intervals consistent with those used by the Vital Statistics of the United States. Maternal age was divided into eight intervals (less than 15, 15–19, . . . , 45 or older) and paternal age into nine intervals (less than 20, 20–24, . . . , 55 or older). Next, the ages of the natural parents at the time of birth of the patient were calculated. Finally, a two-way cross-classification of maternal by paternal age was constructed by assigning each case to the appropriate cell.

Using census data on whites from the Vital Statistics of the United States, the expected joint distribution of paternal and maternal age was constructed by weighting the counts from the appropriate census year by the proportion of NWTS cases born in that year. Expected numbers in each parental age category were computed by normalising the distribution of the total number of Wilms' tumour cases.

Marginal observed and expected counts were obtained by summing over the appropriate row or column of the two-way table. If a cell of one of the marginal tables contained an expected count smaller than two, that cell was combined with an adjacent cell. Marginal relative risks were obtained by taking the ratio of the marginal observed and expected counts (O/E) and dividing by O/E for the lowest age category.

Similar methods were used to compute observed and expected counts for the marginal maternal and paternal age distributions of subgroups determined by these criteria: (1) male or female; (2) bilateral or unilateral tumour; (3) Beckwith-Wiedemann syndrome and/or hemihypertrophy, aniridia, cryptorchidism and/or hypospadias without aniridia, other genitourinary anomalies without aniridia, or no congenital anomaly; (4) intralobar nephrogenic rests, perilobar nephrogenic rests without intralobar rests, or no nephrogenic rests, (5) early (younger than 2 years), middle (2 to 3 years), late (4 to 9 years), or extreme (10 years or older) age-of-onset of tumour, and (6) registration from 1969 to 1978 or from 1979 to 1987. Only patients with synchronous bilateral tumour were included in the bilateral group. All patients not included in one of the four anomaly categories were included in the no congenital anomaly category.

Pearson's chi-squared statistic (Rao, 1965) was used to compare observed and expected counts in the marginal tables. In addition, a $Z$-statistic which compared the observed and expected mean ages was also computed. Expected mean ages $\overline{A}_E$ were computed by weighting the midpoint $A_i$ of each age interval $i$ by the proportion $p_i$ of expected births in that interval ($i = 1, \ldots, K$). Intervals that included the upper or lower tails of the distributions were assigned 'midpoints' 2.5 years above or below the nearest cutpoint, respectively. Observed mean ages $\overline{A}_O$ were computed in a similar fashion. Expected variances were similarly computed using the formula

$$V_E = \sum_{i=1}^{k} p_i \, (A_i - \overline{A}_E)^2$$

so that $Z = (\overline{A}_o - \overline{A}_E)/\sqrt{V_E/N}$, where $N$ is the sample size. $Z$ is normally distributed in large samples even if the parental age distribution is not normal. For small sample sizes, a Wilcoxon signed-rank test was also performed and gave similar results. Because of our prediction that parents of Wilms' tumour patients are older than those in the general population, a one-sided test of hypothesis was employed.

Poisson regression (McCullagh & Nelder, 1990) was used to examine the combined effects of maternal and paternal age on tumour incidence. For this analysis, parental age distributions were partitioned into intervals as described in the beginning of this section. A multiplicative model was fit to the data and expected numbers of cases in each joint interval were used as 'offsets' in the analysis. Likelihood ratio criteria were employed to test for the linear contribution of maternal (paternal) age after paternal (maternal) age was taken into account. Analysis of covariance was used to test for differences in parental age between subgroups, after adjusting for year of birth and year of diagnosis; these analyses were performed separately for maternal and paternal age. Multiple comparisons were performed using Tukey's studentised range test (Neter *et al.*, 1985).

### Results

Of the 4,117 NWTS patients registered from 1969 through 1987, 3,054 were white, born in the US and had no confirmed family history of Wilms' tumour. Paternal age was missing for an additional 600 patients (19.6%) and maternal age for an additional 17 patients, resulting in a final total of 2,437 NWTS patients for the analysis. For the census data, ages were missing for only about 6% of fathers. We assume that the difference reflects additional data missing at random with respect to paternal age. Because census data were unavailable for 1940, 1957, 1958, 1967 and 1968, for the purpose of computing the expected parental age distribution, 42 patients born in 1967 were assumed to have been born in 1966, 70 patients born in 1968 were assumed to have been born in 1969, two patients born in 1957 and 1958 were assumed to have been born in 1959 and one patient born in 1940 was assumed to have been born in 1947.

Marginal observed and expected counts, along with ratios of observed to expected, are given in Table I. Because the expected number of mothers younger than 15 and older than 45 were less than 2 (1.78 and 1.20 respectively), the data in these two cells were combined with those of their respective nearest intervals. Four mothers were younger than 15 and two were older than 45. The Pearson's chi-square statistics and the $Z$ tests comparing observed and expected mean ages suggest a somewhat stronger effect for maternal age.

A plot of paternal age *vs* O/E is shown in Figure 1. For paternal age, O/E increases gradually for young and middle age categories, then more rapidly at older ages. For maternal age, the increase is gradual over the range of age. To exclude the possibility that only the highest age categories contribute to the age effect, a one-tailed $Z$-test was performed after excluding the highest two paternal age categories and was significant ($Z = 2.40$, $P = 0.008$). A similar test excluding the highest maternal age category was also significant ($Z = 2.96$, $P = 0.002$).

Observed and expected counts for the two-way cross-classification of maternal by paternal age categories are given in Table II. Examination of the table suggests that, within most levels of maternal age, O/E increase with paternal age. Similarly, within most levels of paternal age, O/E increases with maternal age. These results suggest that neither maternal nor paternal age alone could explain the deviations of the observed from the expected counts. This question was further examined using Poisson regression. Because of the high correlation between maternal and paternal age, it was not possible to separate the effect of paternal age from that of maternal age.

Results of the subgroup analysis are shown in Table III. A small increase in mean parental age in females is noted. However, analysis of covariance failed to find significant differences between the two groups in either maternal or paternal age; this result is not surprising given the lower power of such an internal analysis compared to the external analysis shown in Table III. Of the 2,437 study cases, nine were missing the indicator for laterality; of the remainder, 132 (5.4%) had bilateral tumour. Although the differences between observed and expected mean parental age appear smaller for the bilateral than for the unilateral group, the differences in the bilateral group are likely to be quite variable due to the much smaller sample size. Analysis of covari-

**Table I** Observed (O) and expected (E) counts for marginal parental age distributions

| | Counts[a] | | | | | | | | | Mean |
|---|---|---|---|---|---|---|---|---|---|---|
| | *<20* | *20–24* | *25–29* | *30–34* | *35–39* | *40–44* | *45–49* | *50–54* | *≥55* | *age[b]* |
| O | 102.00 | 569.00 | 862.00 | 539.00 | 236.00 | 82.00 | 27.00 | 11.00 | 9.00 | 28.94 |
| E | 106.91 | 632.54 | 836.25 | 528.20 | 216.09 | 77.67 | 26.15 | 8.73 | 4.45 | 28.57 |
| O/E | 0.95 | 0.90 | 1.03 | 1.02 | 1.09 | 1.06 | 1.03 | 1.26 | 2.02 | |

[a]$\chi^2_8 = 14.97$, $P = 0.060$. [b]$Z = 2.89$, $P = 0.002$ (one-tailed).

*Maternal age*

| | Counts[a] | | | | | | Mean |
|---|---|---|---|---|---|---|---|
| | *<20* | *20–24* | *25–29* | *30–34* | *35–39* | *≥40* | *Age[b]* |
| O | 243.00 | 821.00 | 844.00 | 381.00 | 121.00 | 27.00 | 26.26 |
| E | 296.00 | 841.50 | 789.96 | 373.62 | 112.55 | 23.37 | 25.93 |
| O/E | 0.82 | 0.98 | 1.07 | 1.02 | 1.08 | 1.16 | |

[a]$\chi^2_5 = 15.03$, $P = 0.010$. [b]$Z = 3.04$, $P = 0.001$ (one-tailed).

**Figure 1** Ratio of observed to expected counts as a function of parental age.

ance failed to find significant differences between the groups when they were compared directly.

Seventy-five (3.1%) of the study cases reported Beckwith-Wiedemann syndrome and/or hemihypertrophy; 18 (0.7%) reported aniridia with or without other anomalies; an additional 64 (2.6%, 5.3% of males) reported cryptorchidism and/or hypospadias; and a further 165 (6.8%) reported other genitourinary anomalies. Sizeable differences between observed and expected ages for mean maternal and paternal age, respectively, were found in most of the anomaly subgroups. A significant and striking increase of almost 2 years in mean paternal age above population expectation was found in patients with cryptorchidism and/or hypospadias. A significant increase of almost 1 year in mean maternal age was found in patients with other genitourinary anomalies. As in the previous analysis, the small sample sizes in these groups suggests caution in interpreting the results, which are likely to be quite variable. The lack of significance of some of the effects may well be due to the small sample sizes rather than a real lack of difference. Analysis of covariance failed to detect significant differences between these groups in the internal comparison.

Of the 1,234 cases that provided sufficient tumour material to detect the presence of nephrogenic rests, intralobar rests were found in 175 (14.2%). A large and significant differences of over 1 year between observed and expected mean paternal age was found as well as moderate effect for maternal age. Perilobar nephrogenic rests were found in 233 of the remaining 1,059 patients. Moderate differences in mean parental age were found for these patients. Analysis of covariance, however, failed to detect significant differences between the groups in the internal comparison.

Patients were also divided into subgroups according to the age of the patient at the time of diagnosis of the tumour. Significant differences between observed and expected parental ages were found for the early and late age-of-onset group. Large differences were also noted in the extreme age-of-onset group. After combining the extreme and late groups, significant differences between the age-of-onset groups were detected using analysis of covariance (maternal: $F_{2,2421} = 5.65$, $P = 0.0036$; paternal: $F_{2,2421} = 3.14$, $P = 0.0433$). Subsequent multiple comparison procedures detected significant differences between the middle and early and between the middle and late groups, but not between the early and late groups.

**Table II** Observed (O) and expected (E) counts for two-way cross-classification of maternal and paternal age

| *Maternal age* | | *Paternal age* | | | | | | | | |
|---|---|---|---|---|---|---|---|---|---|---|
| | | *<20* | *20–24* | *25–29* | *30–34* | *35–39* | *40–44* | *45–49* | *50–54* | *≥55* |
| <20 | O | 86.00 | 119.00 | 30.00 | 6.00 | 2.00 | 0.00 | 0.00 | 0.00 | 0.00 |
| | E | 90.42 | 167.64 | 29.37 | 5.89 | 1.69 | 0.58 | 0.24 | 0.10 | 0.08 |
| | O/E | 0.95 | 0.71 | 1.02 | 1.02 | 1.18 | 0.00 | 0.00 | 0.00 | 0.00 |
| 20–24 | O | 15.00 | 393.00 | 331.00 | 59.00 | 18.00 | 1.00 | 1.00 | 3.00 | 0.00 |
| | E | 15.42 | 414.34 | 326.97 | 62.75 | 14.84 | 4.60 | 1.59 | 0.62 | 0.37 |
| | O/E | 0.97 | 0.95 | 1.01 | 0.94 | 1.21 | 0.22 | 0.63 | 4.87 | 0.00 |
| 25–29 | O | 1.00 | 50.00 | 452.00 | 266.00 | 52.00 | 14.00 | 8.00 | 0.00 | 1.00 |
| | E | 0.88 | 44.92 | 434.04 | 243.41 | 48.24 | 12.30 | 3.99 | 1.41 | 0.78 |
| | O/E | 1.14 | 1.11 | 1.04 | 1.09 | 1.08 | 1.14 | 2.01 | 0.00 | 1.29 |
| 30–34 | O | 0.00 | 7.00 | 45.00 | 190.00 | 102.00 | 24.00 | 5.00 | 4.00 | 4.00 |
| | E | 0.15 | 4.75 | 41.21 | 199.00 | 96.42 | 22.24 | 6.43 | 2.26 | 1.13 |
| | O/E | 0.00 | 1.47 | 1.09 | 0.95 | 1.06 | 1.08 | 0.78 | 1.77 | 3.54 |
| 35–39 | O | 0.00 | 0.00 | 4.00 | 15.00 | 58.00 | 31.00 | 10.00 | 3.00 | 0.00 |
| | E | 0.04 | 0.78 | 4.23 | 16.02 | 51.74 | 28.19 | 7.84 | 2.46 | 1.26 |
| | O/E | 0.00 | 0.00 | 0.94 | 0.94 | 1.12 | 1.10 | 1.28 | 1.22 | 0.00 |
| ≥40 | O | 0.00 | 0.00 | 0.00 | 3.00 | 4.00 | 12.00 | 3.00 | 1.00 | 4.00 |
| | E | 0.01 | 0.10 | 0.43 | 1.12 | 3.16 | 9.77 | 6.06 | 1.87 | 0.84 |
| | O/E | 0.00 | 0.00 | 0.00 | 2.68 | 1.27 | 1.23 | 0.49 | 0.53 | 4.78 |

**Table III** Observed and expected mean ages for various subgroups

| | *Sample* | *Paternal* | | | | *Maternal* | | | |
|---|---|---|---|---|---|---|---|---|---|
| *Subgroup* | *size* | *Observed* | *Expected* | *O-E* | *(Std Err)* | *Observed* | *Expected* | *O-E* | *(Std Err)* |
| Entire sample | 2437 | 28.94 | 28.57 | 0.38[b] | (0.13) | 26.26 | 25.93 | 0.33[b] | (0.11) |
| Male | 1197 | 28.89 | 28.59 | 0.30[a] | (0.18) | 26.19 | 25.95 | 0.24 | (0.16) |
| Female | 1240 | 28.99 | 28.56 | 0.43[b] | (0.18) | 26.33 | 25.92 | 0.41[b] | (0.15) |
| Unilateral | 2296 | 28.94 | 28.57 | 0.37[b] | (0.11) | 26.26 | 25.92 | 0.34[b] | (0.11) |
| Bilateral | 132 | 28.83 | 28.63 | 0.20 | (0.54) | 26.25 | 26.02 | 0.23 | (0.47) |
| No anomaly | 2115 | 28.85 | 28.57 | 0.29[a] | (0.12) | 26.20 | 25.93 | 0.27 | (0.11) |
| B-W/Hemihypertrophy | 75 | 29.30 | 28.49 | 0.81 | (0.73) | 26.23 | 25.84 | 0.39 | (0.62) |
| Aniridia | 18 | 29.17 | 28.60 | 0.56 | (1.48) | 25.83 | 25.96 | −0.12 | (1.27) |
| Cryptorchidism/Hypospadias | 64 | 30.55 | 28.68 | 1.86[b] | (0.78) | 27.03 | 26.09 | 0.94 | (0.67) |
| Other GU | 165 | 29.20 | 28.58 | 0.61 | (0.49) | 26.83 | 25.93 | 0.91[a] | (0.42) |
| No rest | 826 | 28.73 | 28.63 | 0.10 | (0.21) | 26.17 | 26.06 | 0.11 | (0.18) |
| Intralobar ± perilobar | 175 | 30.01 | 28.94 | 1.07[a] | (0.46) | 26.99 | 26.40 | 0.58 | (0.40) |
| Perilobar alone | 233 | 29.05 | 28.66 | 0.38 | (0.40) | 26.56 | 26.10 | 0.45 | (0.35) |
| Early age-of-onset | 807 | 29.18 | 28.71 | 0.47[a] | (0.22) | 26.47 | 26.12 | 0.34[a] | (0.19) |
| Middle age-of-onset | 786 | 28.42 | 28.52 | −0.10 | (0.22) | 25.77 | 25.89 | −0.12 | (0.19) |
| Late age-of-onset | 769 | 29.14 | 28.46 | 0.67[b] | (0.23) | 26.55 | 25.78 | 0.77[b] | (0.20) |
| Extreme age-of-onset | 75 | 29.70 | 28.76 | 0.94 | (0.78) | 26.30 | 25.84 | 0.46 | (0.66) |
| 1969–78 cohort | 803 | 28.84 | 28.40 | 0.44[a] | (0.23) | 26.26 | 25.61 | 0.65[b] | (0.20) |
| 1979–87 cohort | 1634 | 28.99 | 28.66 | 0.33[a] | (0.15) | 26.26 | 26.09 | 0.18 | (0.13) |

[a]$P<0.05$ (one-tailed $Z$ test). [b]$P<0.01$ (one-tailed $Z$ test). Note: $Z$-test compares observed mean age to the mean age of the population.

Finally, patients were divided into two groups based on year of diagnosis. Surprisingly, a large and significant difference between observed and expected maternal age was observed for the 1969–78 cohort but not for the 1979–87 cohort. Significant differences between observed and expected paternal age were observed in both cohorts. In addition, Poisson regression analysis of the 1979–87 cohort shows that paternal age accounts for most of the deviation of observed from expected ages in this cohort; when paternal age is accounted for, there is no difference between observed and expected mean maternal age. Subgroup analysis of the 1979–87 cohort shows results similar to those in Table III, with the notable exception that no parental age differences are found in the early age-of-onset group.

Multiple linear regression analysis was performed to determine if the effect of parental age on any variable could be substantially altered by accounting for the remaining variables. The results (not shown) suggest that congenital anomalies, nephrogenic rests and age-of-onset each make separate contributions, suggesting that these three variables may contribute to the variability in parental age through different mechanisms.

### Discussion

The present study demonstrates a positive relationship between parental age and incidence of sporadic Wilms' tumour, suggesting that at least some Wilms' tumours are the result of new germline mutations in a parent. It should be noted here that ascertainment of US Wilms' tumour cases by the NWTS is not complete. Unascertained cases generally fall in geographical areas served by institutions that do not participate in the NWTS; it is possible that geographical differences in parental age distributions exist which might bias the results.

The difference between the early and late cohorts in the difference between observed and expected maternal age is difficult to explain. One possibility is that the difference observed in the early cohort is an artifact due to sampling bias; it may be that, for whatever reason, older mothers were preferentially included in the NWTS in its early years. The ascertainment probability in the early cohort is 0.37, roughly half that of the later cohort (0.67). On the other hand, the difference may conceivably have been the results of some biological mechanism, such as environmental exposure that is no longer present.

The late cohort is perhaps the more relevant, as it is the more completely ascertained. In this cohort, paternal rather than maternal age is the more important contributor to the parental age effect. This finding is supported by the results of Huff *et al.* (1990), which demonstrate the paternal origin of seven out of eight Wilms' tumour cases with *de novo* constitutional deletions of chromosome 11p13. Risch *et al.* (1987) proposed both linear and exponential 'copy-error' models of paternal mutagenesis and applied them to data on 17 dominant diseases. For diseases displaying a low rate of increase of O/E, they found that (1) both exponential and linear models fit the data equally well, and (2) results were compatible with mutation occurring primarily in males. The size of the effect in the present study is comparable to those in the low-rate-of-increase group of diseases discussed by Risch *et al.* (1987).

The fact that sporadic bilateral Wilms' tumour has earlier average age-of-onset than unilateral tumour (Breslow *et al.*, 1988) is a key tenet of the Knudson-Strong two-hit hypothesis. This study provides no evidence, however, that parents of children with bilateral tumour tend to be older than those of children with unilateral tumour, suggesting that bilateral tumours are no more likely to be the result of new germline mutation in a parent than unilateral tumour. This failure to confirm the predictions of the two-hit model stands in sharp contrast to the finding of Pellie *et al.* (1973) that fathers of patients with sporadic bilateral retinoblastoma are more than 1.2 years older, on average, than father in the general population, while fathers of sporadic unilateral retinoblastoma patients do not differ in average age from fathers in the general population. A similar but less striking result was found for maternal age. This result that lends support to the Knudson-Strong two-hit hypothesis in the case of retinoblastoma is absent in the case of Wilms' tumour.

Evidence is found that parental ages of patients with at least some associated congenital anomalies are higher than expected. Also, parents of patients with rest-associated tumours are substantially older than parents of the remaining patients. In particular, fathers of patients with intralobar nephrogenic rests are, on average, more than a year older than expected, fathers of patients with cryptorchism and/or hypospadias are almost 2 years older than expected, and mothers of patients with other genitourinary anomalies are almost 1 year older than expected. This combination of patient/tumour characteristics is associated with mutations in the 11p13 region (e.g. Pelletier *et al.*, 1991; Pritchard-Jones & Fleming, 1991) and suggests that many tumours which involve mutations to the WT1 gene may be the result of new germinal mutations.

The observation of a parental age effect for early age-of-onset patients is not surprising, as patients with intralobar nephrogenic rests and/or genitourinary anomalies or aniridia tend to have early age-of-onset (Breslow *et al.*, 1988). However, patients with perilobar nephrogenic rests and/or Beckwith-Wiedemann syndrome or hemihypertrophy do not differ in age-of-onset distribution from the main body of patients (Breslow *et al.*, 1988). The presence of strong parental age differences in late age-of-onset patients may be indicative of the presence of a subtype of Wilms' tumours other than those associated with Beckwith-Wiedemann syndrome and genitourinary anomalies and aniridia.

It has been hypothesised that the presence of nephrogenic rests reflect events early in development (Beckwith *et al.*, 1990). In particular, the presence of intralobar nephrogenic rests are believed to indicate an earlier developmental disturbance than the presence of perilobar nephrogenic rests. The finding of parental age effects in these subgroups suggests that some of the relevant events occur prior to conception. If so, routine identification of rest-associated disease may prove to be helpful in diagnosis of the etiologic origin of Wilms' tumours and, consequently, assessment of risk to relatives of patients. A better understanding of these issues will come from a study of the increasing numbers of survivors and their offspring. Two such studies (Li *et al.*, 1987; Mulvihill *et al.*, 1987) have found only one case of Wilms' tumour among 225 offspring of childhood kidney tumour survivors. It is hoped that continued follow-up of the large NWTS series, with its increasing numbers of survivors approaching the childbearing years, will help resolve some of these issues.

This work was supported by grants 1-R01-CA54498 and 5-T32-CA09168-17 from the National Institutes of Health. The authors thank the many pathologists, surgeons, pediatricians, radiation therapists and other health professionals of the Pediatric Oncology Group and Children's Cancer Study Group who managed these children, without whom this study would have been impossible; the staff of the NWTS Data and Statistical Center, for their invaluable assistance; and members of the NWTS Committee for their helpful advice.

### References

BECKWITH, J.B., KIVIAT, N.B. & BONADIO, J.F. (1990). Nephrogenic rests, nephroblastomatosis, and the pathogenesis of Wilms' tumor. *Pediatr. Pathol.*, **10**, 1-36.

BRESLOW, N., BECKWITH, J.B., CIOL, M. & SHARPLES, K. (1988). Age distribution of Wilms' tumor: report from the National Wilms' tumor study. *Cancer Res.*, **48**, 1653-1657.

COMINGS, D.E. (1973). A general theory of carcinogenesis. *Proc. Natl Acad. Sci. USA*, **70**, 3324-3328.

COPPES, M.J., BONETTA, L., HUANG, A., HOBAN, P., CHILTON-MACNEILL, S., CAMPBELL, C.E., WEKSBERG, R., YEGER, H., REEVE, A.E. & WILLIAMS, B.R.G. (1992). Loss of heterozygosity mapping in Wilms' tumor indicates the involvement of three distinct regions and a limited role for non-disjunction or mitotic recombination. *Genes, Chromosomes & Cancer* (in press).

GLOECKLER, L. (1992). Personal communication.

HUFF, V., MEADOWS, A., RICCARDI, V.M., STRONG, L.C. & SAUNDERS, G.F. (1990). Parental origin of de novo constitutional deletions of chromosomal band 11p13. *Am. J. Hum. Genet.*, **47**, 155-160.

KNUDSON, A.G. & STRONG, L.C. (1972). Mutation and cancer: a model for Wilms' tumor of the kidney. *J. Natl Cancer Inst.*, **48**, 313-324.

LI, F.P., GIMBERE, K., GELBER, R.D., SALLAN, S.E., FLAMANT, F., GREEN, D.M., HEYN, R.M. & MEADOWS, A.T. (1987). Outcome of pregnancy in survivors of Wilms' tumor. *J. Amer. Med. Assoc.*, **257**, 216-219.

MANNENS, M., SLATER, R.M., HEYTING, C., DE KRAKER, J., COAD, N., DE PAGTER-HOLTHUIZEN, P. & PEARSON, P.L. (1988). Molecular nature of genetic changes resulting in loss of heterozygosity of chromosome 11 in Wilms' tumours. *Hum. Genet.*, **81**, 41-48.

MCCULLAGH, P. & NELDER, J.A. (1990). *Generalized Linear Models*, 2nd Ed., pp. 193-209. London: Chapman and Hall.

MULVILHILL, J.J., MYERS, M.H., CONNELLY, R.R., AUSTIN, D.F., BRAGG, K., COOK, J.W., HASSINGER, D.D., HOLMES, F.F. & HOLMES, G.F. (1987). Cancer in offspring of long-term survivors of childhood and adolescent cancer. *Lancet*, **2**, 813-817.

NETER, J., WASSERMAN, W. & KUTNER, M.N. (1985). *Applied Linear Statistical Models*, 2nd Ed., pp. 574-579. Homewood, Illinois: Richard D. Irwin.

PAL, N., WADEY, R.B., BUCKLE, B., YEOMANS, E., PRITCHARD, J. & COWELL, J.K. (1990). Preferential loss of maternal alleles in sporadic Wilms' tumour. *Oncogene*, **5**, 1665-1668.

PELLETIER, J., BRUENING, W., LI, F.P., HABER, D.A., GLASER, T. & HOUSMAN, D.E. (1991). WT1 mutations contribute to abnormal genital system development and hereditary Wilms' tumour. *Nature*, **353**, 431-434.

PELLIE, C., BRIARD, M.-L., FEINGOLD, J. & FREZAL, J. (1973). Parental age in retinoblastoma. *Humangenetik*, **20**, 59-62.

PRITCHARD-JONES, K. & FLEMING, S. (1991). Cell type expressing the Wilms' tumour gene (WT1) in Wilms' tumours: implications for tumour histogenesis. *Oncogene*, **6**, 2211-2220.

RAO, C.R. (1965). *Linear Statistical Inference and Its Applications*, pp. 325–330. New York: John Wiley and Sons.

RICCARDI, V.M., DOBSON II, C.E., CHAKRABORTY, R. & BONTKE, C. (1984). The pathophysiology of neurofibromatosis: IX. Paternal age as a factor in the origin of new mutations. *Am. J. Med. Genet.*, **18**, 169–176.

RISCH, N., REICH, E.W., WISHNICK, M.M. & MCCARTHY, J.G. (1987). Spontaneous mutation and parental age in humans. *Am. J. Hum. Genet.*, **41**, 281–248.

VOGEL, F. & RATHENBERG, R. (1975). Spontaneous mutation in man. *Adv. Hum. Genet.*, **5**, 223–318.